# Broadcast Authentication for Wireless Sensor Networks Using Nested Hashing and the Chinese Remainder Theorem

**DOI:** 10.3390/s100908683

**Published:** 2010-09-17

**Authors:** Mohamed Hamdy Eldefrawy, Muhammad Khurram Khan, Khaled Alghathbar, Eun-Suk Cho

**Affiliations:** 1 Center of Excellence in Information Assurance (CoEIA), King Saud University, PO Box 92144, Riyadh 11653, Saudi Arabia; E-Mails: meldefrawy@ksu.edu.sa (M.H.E.); kalghathbar@ksu.edu.sa (K.A.); 2 Information Systems Department, College of Computer and Information Sciences, King Saud University, Saudi Arabia; 3 Department of Multimedia, Hannam University, 133 Ojeong-dong, Daedeok-gu, Daejeon 306-791, Korea; E-Mail: eunsukk@empal.com (E.S.C.)

**Keywords:** wireless sensor network, authenticated broadcast, nested hashing chains, Chinese Remainder Theorem

## Abstract

Secure broadcasting is an essential feature for critical operations in wireless sensor network (WSNs). However, due to the limited resources of sensor networks, verifying the authenticity for broadcasted messages is a very difficult issue. μTESLA is a broadcast authentication protocol, which uses network-wide loose time synchronization with one-way hashed keys to provide the authenticity verification. However, it suffers from several flaws considering the delay tolerance, and the chain length restriction. In this paper, we propose a protocol which provides broadcast authentication for wireless sensor networks. This protocol uses a nested hash chain of two different hash functions and the Chinese Remainder Theorem (CRT). The two different nested hash functions are employed for the seed updating and the key generation. Each sensor node is challenged independently with a common broadcasting message using the CRT. Our algorithm provides forward and non-restricted key generation, and in addition, no time synchronization is required. Furthermore, receivers can instantly authenticate packets in real time. Moreover, the comprehensive analysis shows that this scheme is efficient and practical, and can achieve better performance than the μTESLA system.

## 1. Introduction

Achieving broadcast security is a must for wireless sensor networks; hence it is necessary for the base station to broadcast commands and data to sensor nodes. Without secure communication, sensors may be involved in incorrect operations and can’t meet the network requirements. The current security solutions for wired and wireless networks cannot be utilized for a wireless sensor network because of the energy, memory and computation restrictions of the latter. These limitations make the design and operation completely dissimilar to those of regular wireless networks. Broadcast authentication based on asymmetric key cryptography cannot deal with the limited resource constrains. Symmetric key cryptography and hash functions are cheaper in their computational requirements and are more widely utilized in sensor networks [1,2]. WSNs’ broadcast authentication was first covered by TESLA [3], and μTESLA [4] that provides the asymmetric cryptographic property of authenticated broadcast through delayed disclosing (time-varying) of symmetric keys. The base-station installs a key chain by repeatedly applying a one way hash function (OWHF) to an initial random value, called seed. The chain construction allows nodes to verify the authenticity of the disclosed keys. Loosely time synchronized and MAC (Message Authentication Code) generations are required. Revelation of session keys by the base-station is delayed, thus allowing nodes to verify the key validity.

Multilevel μTESLA [5] is proposed to reduce the need to reinitialize the network by implementing multiple levels of key chains, in which high-level keys are used to communicate root-keys (or commitments) for low-level chains, which are used in turn for broadcast authentication as in standard μTESLA. Network lifetime is extended. Significant computation and storage are required. Receivers can’t deal with the received messages instantly and have to store them within one or several time intervals. Considering the broadcasting of urgent messages like alerts and alarms; the TESLA family has great shortcomings in dealing with such matters. Furthermore, the delayed authentication can be subject to Denial-of-Services (DoS) attacks. Merkle tree utilization [6] was introduced to overcome this shortage in bandwidth and storage resources utilization. TIK [7] was proposed to achieve immediate authentication based on sensitive time synchronization between the sink and the receiving nodes. However, this technique is not suitable for WSNs, as mentioned by its inventors. Sensor nodes have a limited battery life, which can make using asymmetric key techniques impractical as they use much more energy for their mathematical calculations. We propose a new algorithm that uses two different types of hash functions, which come with a nested chain and the Chinese Reminder Theorem in order to get a common broadcasting message. The resulting chain provides the forwardness and the infiniteness, and no process restarting is required. The proposed protocol is compared with others in terms of its computational cost and security attributes.

The rest of this paper is organized as follows: Section 2 discusses the related work, Section 3 discuses the required attributes, Section 4 proposes our new algorithm, Section 5 evaluates our scheme’s performance, Section 6 analyzes the security attributes, and finally Section 7 concludes the paper.

## 2. Related Work

The following subsection discuses some of the schemes related to WSN authentication broadcasting. Their efficiency and shortcomings according to the desirable security attributes that will be discussed will also be illustrated.

### 2.1. Lamport’s Scheme

Hash chains were first proposed by Lamport [8]. They involve applying a hash function *h*(·) *N* times to a seed (*s*) to form a hash chain of length *N*:

(1) $$h^{1}(s),h^{2}(s),\ldots ,h^{N-1}(s),h^{N}(s)$$

The user calculates the *i*-th key according to this relation:

(2) $$k_{i}(s)=h^{N-i}(s)$$

The host authenticates the user by checking that the following equality holds:

(3) $$h(k_{t}(s))=h^{N-i+1}(s)$$

where the value *h*^*N*−*i*+1^(*s*) is already saved in the host system’s file from the previous *i*-th authentication. After any successful authentication, the system password file is updated with the new key. This scheme has a limitation on the number of authentications, so that after reaching *N* authentications, a process restart is required. In addition, it is vulnerable to an opponent who sends small challenge values to users that respond with the chain initial values [9]. This attack can be referred to as a small challenge attack. Also, the users are charged with computational processes through the initialization phase, which makes the system unsuitable for WSNs.

### 2.2. Bicakci et al.’s Scheme

The infinite length hash chains (ILHC) proposed by [10] use a public-key algorithm, *A*, to produce a forward and infinite one way function (OWF). Bicakci *et al*. utilized RSA [11], where d is the private key and e is the public key. The OTP originating from initial input “*s*” using the RSA public-key algorithm for the *i*-th authentication is:

(4) $$k_{i}(s)=A^{i}(s,d)$$

and the verification of the *i*-th key is done by:

(5) $$k_{i-1}(s)=A\;(k_{i},e)$$

increasing the number of cascaded exponentiations increases the computational complexity, making this algorithm very difficult to implement in limited computation devices [12].

### 2.3. Chinese Remainder Theorem (CRT)

If the integers *n*_1_, *n*_2_,…,*n_k_* are pair-wise relatively prime, then the system of simultaneous congruence:

(6) $$\begin{array}{c} x\equiv r_{1}\;\text{mod}\;n_{1}\\ x\equiv r_{2}\;\text{mod}\;n_{2}\\ \vdots \\ x\equiv r_{k}\;\text{mod}\;n_{k} \end{array}$$

has a unique solution: $x=\sum_{i=1}^{k}r_{i}N_{i}^{-1}N_{i}\;\text{mod}\;N$ where;

(7) $$N=\prod_{i=1}^{k}n_{i}$$

(8) $$N_{i}=\frac{N}{n_{i}}$$

(9) $$N_{i}^{-1}N_{i}\equiv 1\;\text{mod}\;n_{i}$$

### 2.4. TESLA Family Broadcast Authentication

Timed Efficient Stream Loss-tolerant Authentication (TESLA) [3] is a multicast stream authentication protocol. Keys used to authenticate the *i*-th message is disclosed along with *(i + 1)*-th message. μTESLA [4] provides authentication for data broadcasts, and requires that base station and sensor nodes be loosely time synchronized. According to Lamport’s scheme, a base station (BS) randomly selects the last key *k_n_*, the chain seed, and applies a one-way public function *h*(·) to generate the rest of keys: *k*_0_, *k*_1_,..., *k_n_*_−1_ as *k_i_* = *h*(*k_i_*_+1_). Given *k_i_*, every sensor node can generate the sequence *k*_0_, *k*_1_, ..., *k_n_*_−1_. However, given *k_i_*, no one can generate *k_i_*_+1_. At *i*-th time slot, BS sends an authenticated message *MAC_k_i__* (*message*). Sensor nodes store the message till the verification key in the *(i + 1)*-th time slot is disclosed. Sensor nodes verify disclosed key *k_i_*_+1_ by using key *k_i_* as *k_i_* = *h*(*k_i_*_+1_). In μTESLA, nodes are required to store a message until the authentication key is disclosed. This operation may create storage problems, and encourages DoS types of attacks.

μTESLA has been expanded to Multi-level μTESLA [4] by simplifying the key distribution phase and introducing a new concept of a multi-level key chain generation using pseudo-random functions that improves the protocol efficiency. Multi-level μTESLA reduces the need to reinitialize the network (although re-initialization is still required) by implementing multiple levels of key chains, in which high-level keys are used to communicate root-keys (or commitments) for low-level chains which are used in turn for broadcast authentication as in standard μTESLA. The chains are further connected in that each root-key is derived from the corresponding high-level chain using another pseudo-random function. Network lifetime is extended many times over, but it is still limited. A problem would result if a receiver dropped a related commitment distribution message initializing a new low-level chain; it would be unable to verify any broadcast data received during this entire lifetime of the chain itself. The data would still be verifiable eventually as the receiver could use any later commitment distribution message to reconstruct all the lost high-level keys and the corresponding chains. This would require significant computation and storage.

### 2.5. CRTBA Broadcast Authentication

The scheme proposed in [13] is divided into three phases: Distribution, Message Signing, and finally Message Authentication phase. Before deployment all nodes are loaded with the chain seed, *k_n_*, the OWHF *h*(·), and two different modules values, *n_A_* and *n_B_* for the CRT. When the BS needs to broadcast a message *m* to sensor nodes for the *i*-th session, BS calculates the MAC of the message m using *k_i_* to get *M* = *MAC_k_i__* (*m*). After that BS cipher *k_i_* and *M* using the two secrets values *n_A_* and *n_B_* through the CRT to get: *U* ≡ *k_i_* mod *n_A_* and *U* ≡ *M* mod *n_B_*, then it broadcast *U*. Upon the occurrence of *U* reception by sensor nodes, they recover *k_i_* from *U*, and then apply the OWHF *h*(·), to check $k_{j}\overset{?}{=}h^{i-j}(k_{i})$ where *k_j_* is the last authentic key that sensor nodes have received. Finally, to verify the message integrity, the sensor nodes compute the corresponding MAC using *k_i_* of the received message and then compare the result. Unfortunately, this scheme also has a length restriction considering the use of a backward hashing chain to generate keys.

## 3. Required Attributes

Here we list a number of desirable security attributes for authenticated broadcast:

### 3.1. Data Integrity

Data integrity ensures that data has not been altered by unauthorized entities.

### 3.2. Data Origin Authentication

Data Origin Authentication guarantees the origin of data. It is a fundamental step in achieving entity authentication in protocols as well as establishing keys. We may say that data origin authentication implies data integrity. So it is not possible to achieve data integrity without data origin authentication.

### 3.3. Freshness

Packets that have been captured and replayed at a later time should be ignored by the sensor nodes.

### 3.4. Delay Tolerance

No time synchronization should be required in the system for data verification. Each packet must be verifiable without having to wait for additional data.

### 3.5. Confidentiality

Confidentiality ensures that data is only available to those authorized to obtain it.

### 3.6. Denial-of-Service Attack

The denial of service attack is an attempt to make a node resource unavailable to its intended users.

### 3.7. Small Challenge Attack

This attack challenges the backward hashing with small values to respond with the chain initial values.

### 3.8. Limitation for an N times Authentications

Process re-initialization after *N* of authentications is necessary.

## 4. Our Approach

The basic idea of our scheme is to expand Lamport’s scheme [8] with some modifications that produce the desirable infiniteness and forwardness, avoiding the use of public key cryptography. The shortcoming of those two parameters, infiniteness and forwardness, causes the insufficiency shown with respect to the previous work.

**Figure 1.**
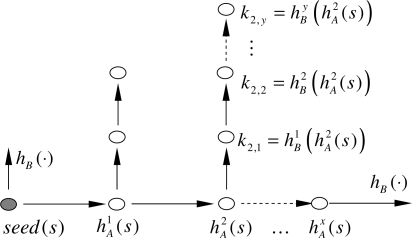
Session key production considering a nested hash chain using two different hashes.

Thus we need to integrate Lamport’s scheme using two different one way hash functions, *h_A_*(·) and *h_B_*(·), one for the seed chain and the other for the session key’s production, as shown in Figure 1.

### 4.1. Key Pre-loading Phase

Each node *n_j_* is loaded with two unique CRT modules $r_{n_{j}}^{A}$ and $r_{n_{j}}^{B}$. Those modules, regarding the all nodes, are relatively primes. Also all sensors are loaded with key seed〈*s*〉 and the two different hash functions, *h_A_*(·) and *h_B_*(·). From the other way the base station is loaded with all this information considering the all the CRT modules for all the network’s nodes, the key seed〈*s*〉, and the two different hash functions *h_A_*(·) and *h_B_*(·).

### 4.2. Message Authentication

Before the broadcasting operation, BS has to do the following:

- Calculate the session key $k_{x_{i},y_{i}}=h_{B}^{y_{i}}\left(h_{A}^{x_{i}}(s)\right)$ for the *i*-th authentication.
- Encrypt the broadcasted message *m* concatenated with the session key *k_x_i_, y_i__* with the session key to get *U* = *E_k_x_i_,y_i___* (*m*‖*k_x_i_,y_i__*)
- Calculate the broadcasted chain indexes, *X*, for the all *N* nodes considering the CRT
  (10) $$\begin{array}{c} X\equiv x_{i}\;\text{mod}\;r_{n_{1}}^{A}\\ X\equiv y_{i}\;\text{mod}\;r_{n_{1}}^{B}\\ X\equiv x_{i}\;\text{mod}\;r_{n_{2}}^{A}\\ X\equiv y_{i}\;\text{mod}\;r_{n_{2}}^{B}\\ \vdots \\ X\equiv x_{i}\;\text{mod}\;r_{n_{j}}^{A}\\ X\equiv y_{i}\;\text{mod}\;r_{n_{j}}^{B}\\ \vdots \\ X\equiv x_{i}\;\text{mod}\;r_{n_{N}}^{A}\\ X\equiv y_{i}\;\text{mod}\;r_{n_{N}}^{B} \end{array}$$

The BS constructs the broadcasted packet to be *P_i_* = {*E_k_x_i_,y_i___* (*m*‖*k_x_i_,y_i__*)‖*X*} and then broadcast it to all sensors.

### 4.3. Authentication Verification

Upon the reception of *P_i_* by the all sensors, they will need to ensure that the broadcast packets come from the authenticated BS. The verification process is done as follows:

- Each sensor node will extract *X* to perform the module operation to obtain the chain indexes, e.g., *n*_1_ will get $x_{i}\equiv X\;\text{mod}\;r_{n_{1}}^{A}$ and $y_{i}\equiv X\;\text{mod}\;r_{n_{1}}^{B}$.
- After getting the chain indexes, they will perform the key generation according to these indexes by using the two different hash functions to get this $k_{x_{i},y_{i}}=h_{B}^{y_{i}}\left(h_{A}^{x_{i}}(s)\right)$.
- By decrypting D*_k_x_i_,y_i___* (*U*), sensors will be able to get the message m and the session key *k_x_i_,y_i__*.
- Then the sensor nodes need to compare the two sessions they have established and received, if the comparison is positive, then sensor nodes will recover the message. Otherwise the received broadcast message has been altered. The message integrity also checked implicitly through the authentication verification, that way tampering with *U* in a way of message modification will sequentially affect the received session key.
- After the completion of one session, sensor nodes and BS have to update the current seed to the next one:
  (11) $$s_{\mathrm{nxt}}=\left(h_{A}^{x_{i}}(s_{\mathrm{crt}})\right)$$

## 5. Performance Analysis

In this section, we are going to analyze the performance of our algorithm with respect to the storage and computational cost [14].

### 5.1. Storage Analysis

The storage complexity is the amount of memory (RAM size) required to store security credentials. The storage complexity affects the hardware price of sensor nodes. Our proposal requires the base station to save two keys for each sensor nodes to build the conference *X*, two different hash functions *h_A_*(·) and *h_B_*(·), and one seed〈*s*.〉 This storage overhead is neglected to the base station, since the base station regarded as resource-rich node. In the other way, sensor node *n_j_* has to store two privet keys $r_{n_{j}}^{A}$ and $r_{n_{j}}^{B}$, and one seed 〈*s*〉, each one of them is 160-bit. This tells us that the memory required for credentials per module (RAM) is 160 × 3-bit = 480-bit = 60-bytes. Hash functions *h_A_*(·) and *h_B_*(·) are implemented, written in nesC code for TinyOS, in approximately 20 Kbyte of memory (ROM.)

### 5.2. Computation Analysis

Considering the computational complexity, base station has to build the congruent equation (10) to reach the chain indexes for all sensors, *X*, also it has to perform two different hash operations to build the session key *k_x_i_,y_i__* this computation is affordable in the base station. Alternatively sensor nodes have to do two different modulo operation and to perform the same two different hash operations according to *h_A_*(·) and *h_B_*(·). This also is very easy to the sensor nodes. Rather than the previous techniques which use backward hash functions. Those previous techniques cost the sensor nodes to perform hashing operations for many times, especially through the chain initial values.

**Example:** Considering the chain length to be N = 1,000 the number of required hash operation considering Lamport scheme will be. (N + 1) × (N/2) = 500,500. On the contrary the usage of nested hashing will require the sensors to perform 2*N* hash operations which are equal to 2,000, according to our illustration. This could show how the nested hashing using two different hash chains is very cheap, in a very simple way.

Now, we consider the required execution time for a sensor node to calculate the session key $k_{x_{i},y_{i}}=h_{B}^{y_{i}}\left(h_{A}^{x_{i}}(s)\right)$. The utilization of the microprocessor Sparc(400) as the sensor nodes’ platform, will give us the following: the required time to digitize a plain text of size 80 bytes using MD5 will cost us a = 39 μs and also, the required time to digitize a plain text of size 64 bytes using SHA-1 will cost us b = 56 μs as shown in Table 2 [15], such that the total time required to calculate the session key $k_{x_{i},y_{i}}=h_{B}^{y_{i}}\left(h_{A}^{x_{i}}(s)\right)$ is *t_exec_* = *a*×*x_i_* + *b*×*y_i_*. Considering that the maximum values for *x_i_* and *y_i_* are *w* = 10, hence t_exec_ = 10(56 + 39) = 0.95 ms. Note we have considered the worst case, hence we have considered the largest input plaintext for the both two hash algorithms, but in fact the plain text size will be no more than 160-bits = 20-bytes, rather than the 80 bytes or 64 bytes.

However, the time required for individual modulo operations $\text{mod}\;r_{n_{j}}^{A}$ and $\text{mod}\;r_{n_{j}}^{B}$ for node *n_j_* is tiny compared to the calculation of the two different hash operations.

## 6. Security Analysis

According to the security attributes we have mentioned above, we are going to evaluate our approach:

### 6.1. Data Integrity

An implicit check for data integrity has been provided. Any data modifications that could be done will consequently affect the received vector *U* = *E_k_x_i_,y_i___* (*m*‖*k_x_i_,y_i__*) which will be discovered through the key checking, by comparing the two sessions they have established and received.

### 6.2. Data Origin Authentication

Sending an original copy of the session key concatenated with the message and then encrypting them with the same key provides the originality authentication in a straightforward way. No one has the ability to build the broadcasted packet *P_i_* = {*E_k_x_i_,y_i___* (*m*‖*k_x_i_,y_i__*)‖*X*} except for the base-station or an intruder that has captured the entire congruence keys $r_{n_{j}}^{A}$ and $r_{n_{j}}^{B}$ for all nodes. This broadcast message has to provide the positivity authentication check considering the all sensor nodes.

### 6.3. Freshness

Our proposal allows the base station to challenge the sensor nodes with unpredictable uniformly distributed values of (x_i_, y_i_). According to these values, and according to the seed updating every session, new refreshed keys have been established every session, so the communication system has a new and refreshed session key, and previous messages cannot be replayed. If we suppose that x_i_ and y_i_ can take one value of forward m values, the probability of successfully guessing a challenge will be the joint probability of x_i_ and y_i_, which is equal to 1/m^2^. We can refer to this property as the ability to resist predictable attacks.

### 6.4. Delay Tolerance

Our proposed scheme provides an instant authentication. Every broadcasted packet contains the authentication information for itself, independently of previous and following messages. The authentication process is done in the same session.

### 6.5. Confidentiality

Confidentiality cannot be guaranteed if one or more nodes have been compromised. If an intruder acquires the ability to capture one node or more he will be able to solve the congruent equation using the captured node *n_j_* congruent keys $r_{n_{j}}^{A}$ and $r_{n_{j}}^{B}$. The CRTBA [13] algorithm also did not cover this property, furthermore the broadcasted messages are sent in the plain form without encryption. Actually, regarding certain applications like the broadcasting of urgent alert notifications and warning systems need instant message authentication rather than confidentiality.

### 6.6. Denial of Service Attacks

In μTESLA scheme, the sensor nodes can’t authenticate the received message immediately after reception. The intruder can send a large amount of forged messages to consume the sensor nodes buffer. The instant authentication provided in our scheme, overcomes this weakness. The authentication process is done in the same session independently of the previous or the next sessions. This vulnerability is overcome without resources an extra bandwidth or an extra storage memory like [5] and [6].

### 6.7. Limitation for an N times Authentications

All TESLA families and also CRTBA, use backward hash chain. The backward chain has a restriction of an *N* time for authentications; a process restart is required after reaching this number of authentications. Our algorithm utilizes a new technique of employing two nested and different hash functions for the key production. This technique uses forward hashing and has no need for process restarting after reaching any number of authentications.

### 6.8. Small Challenge Attack

Utilizing a one way hash function to construct a hashing chain in the backward fashion encourages a new kind of attack called small challenge attack. This type of attack discloses the hash chain initial values. These initial values help the intruder to extract the remaining chain values by hashing those initial values. Our algorithm covers this vulnerability by the utilization of two different and nested hash functions in the forward fashion, which prevents this kind of attack.

### 6.9. Brute Force Attack

The ability of generating a truly random sequence of key bits can defeat a brute force attack, as a brute force attack would have no way of distinguishing one key from the other. Relying on the generation of random number can impede the brute force. The nested hashing progress random values for *i*-th authentication (x_i_, y_i_). play a great role in preventing this type of attacks according to the entropy of their random generation.

## 7. Conclusions

A new wireless sensor network broadcast authentication scheme based on forward hashing using two different nested hashes and the Chinese Reminder Theorem (CRT) has been presented. The broadcasting messages are built using the congruence of the CRT. The two different hashing systems are utilized in the session key generation in a forward and unlimited way. This scheme achieves better characteristics than the other schemes, we discussed. Our proposal is not limited to a certain number of authentications, and also does not involve computationally expensive techniques (PKC) to provide infiniteness. A detailed security analysis has been performed that covers many types of attacks that could influence our scheme. Our scheme satisfies all the security attributes, we have discussed, except for the confidentiality in case of one node or more has been captured. This scheme is applicable for alerting and warning systems that need instant broadcast authentication rather than message confidentiality.

## Figures and Tables

**Figure 2.**
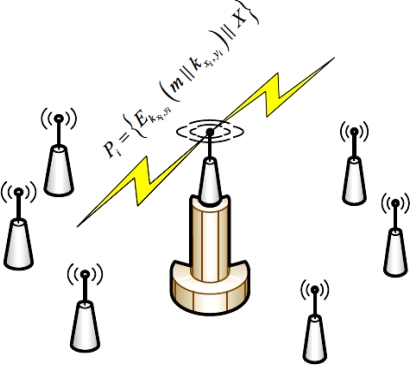
The Proposed Broadcasting Authentication Scheme in Wireless Sensor Network.

**Table 1.** The Proposed Scheme Notation.

**Notation**	**Description**
*h_A_* (·)	Represents the first hash function
*h_B_* (·)	Represents the second hash function
(*x_i_*, *y_i_*)	The nested hashing progress values for *i*-th authentication
$h_{B}^{y_{i}}\left(h_{A}^{x_{i}}(s)\right)$	Hashing the seed by *h_A_* (·) for *x_i_* times followed by *h_B_* (·) hashing for *y_i_* times for the *i*-th session
*k_x_i_, y_i__*	Session key for the *i*-th authentication
*U*	The encryption of the concatenated message with the session by the session key
*P_i_*	The podcasted packet for the *i*-th authentication
*X*	The broadcasted chain indexes, calculated by the CRT
*S_crt_*	The current seed
*S_nxt_*	The next seed

**Table 2.** Execution times [*μs*] for two different hash algorithms, platforms and plaintext sizes [bytes].

**Algorithm**	**Size**	**Atmega103**	**Atmega128**	**M16C/10**	**StrongARM**	**Xscale(400)**	**Xscale(200)**	**Sparc(440)**
MD5	0	5,863	1,466	1,083	46	26	53	23
	1:26	5,890	1,473	1,075	46	26	53	23
	62:80	10,888	2,722	2,011	74	45	90	39
SHA-1	1	15,249	3,812	2,651	69	51	102	27
	3	15,781	3,945	5,303	69	50	103	27
	65	14543	3636	7955	133	102	205	55
	64	31,107	7,777	10,907	145	103	207	56
